# Total Number of Alterations in Liquid Biopsies Is an Independent Predictor of Survival in Patients With Advanced Cancers

**DOI:** 10.1200/PO.19.00204

**Published:** 2020-03-24

**Authors:** Peter Vu, Yulian Khagi, Paul Riviere, Aaron Goodman, Razelle Kurzrock

**Affiliations:** ^1^ Center for Personalized Cancer Therapy and Division of Hematology and Oncology, Department of Medicine, University of California San Diego Moores Cancer Center, La Jolla, CA; ^2^ Division of Blood and Marrow Transplantation, Department of Medicine, University of California San Diego Moores Cancer Center, La Jolla, CA

## Abstract

**PURPOSE:**

Studies have demonstrated an association between quantity of circulating tumor DNA (ctDNA) and poorer survival. We investigated the relationship between percent ctDNA (%ctDNA), total number of ctDNA alterations, and overall survival (OS) in liquid biopsies.

**MATERIALS AND METHODS:**

Overall, 418 patients with blood-based next-generation sequencing (54 to 73 genes) were analyzed. Eligible patients included those who had advanced/metastatic solid tumor malignancies and never received immunotherapy treatment, which may alter the survival curve in patients with high mutational burden.

**RESULTS:**

Patients with a high (≥ 5%) %ctDNA had significantly shorter OS versus those with intermediate (≥ 0.4% to < 5%) or low (< 0.4%) values (median OS, 7.0 *v* 14.1 *v* not reached [NR] months, respectively; *P* < .0001). Patients with a high (≥ 5) total number of alterations had significantly shorter OS versus those with intermediate (≥ 1.46 to < 5), low (< 1.46), or no alterations (median OS, 4.6 *v* 11.7 *v* 21.3 *v* NR months, respectively; *P* < .0001). The total number of alterations correlated with %ctDNA (r = 0.85; 95% CI, 0.81 to 0.87; *P* < .0001). However, only an intermediate to high total number of alterations (≥ 1.46) was an independent predictor of worse OS (hazard ratio, 1.96; 95% CI, 1.30 to 2.96; *P* = .0014; multivariate analysis).

**CONCLUSION:**

We demonstrate that the total number of alterations and %ctDNA have prognostic value and correlate with one another, but only the total number of alterations was independently associated with survival outcomes. Our findings suggest that the total number of alterations in plasma may be an indicator of more aggressive tumor biology and therefore poorer survival.

## INTRODUCTION

Five-year survival rates are incredibly variable among cancer types, ranging from more than 90% in prostate cancer to less than 8% in pancreas cancer, and depend heavily on clinical and pathologic stage.^1^ Although repeat tissue biopsies during the course of treatment or at the time of progression may provide clinically important information, such biopsies are not routinely performed because they can be technically difficult, time consuming, medically invasive, and lead to complications. However, liquid biopsies, or cell-free DNA (cfDNA) obtained from blood plasma that contains fragments of circulating tumor DNA (ctDNA) shed from tumor cells into the bloodstream, can identify new actionable alterations and be performed repeatedly with minimal procedural risk.^2-5^ ctDNA can then be analyzed using technologies such as digital polymerase chain reaction to detect specific known somatic variants (eg, *EGFR* T790M) or next-generation sequencing (NGS) that uses massive parallel sequencing to detect up to thousands of somatic and germline alterations in a single run.^6^ In addition, genomic alterations found on liquid biopsies are often concordant with alterations found on tissue biopsy when obtained within close proximity to one another.^7-9^

A number of studies have demonstrated that there is an association between higher amounts of cfDNA or ctDNA and poorer survival, perhaps because percent ctDNA (%ctDNA) correlates with tumor burden.^10,11^ For the most part, these reports dichotomized the level of cfDNA or ctDNA at a cut-point (often but not always at approximately 5% or 10% ctDNA).^10,12-18^ In the case of surgical candidates, the cut-points may be lower. For instance, Baumgartner et al^15^ found that preoperative levels of %ctDNA ≥ 0.25% in patients with peritoneal carcinomatosis were an independent predictor of shorter progression-free survival. In the current study, we sought to more comprehensively examine the relationship between %ctDNA versus the total number of alterations found in liquid biopsies and outcome.

**Context**

**Key Objective**
Can we use liquid biopsies to obtain prognostic information for patients with advanced cancers?
**Knowledge Generated**
We demonstrate that an increasing number of genomic alterations found on liquid biopsy correlates with progressively worse survival in patients with GI and other advanced cancers, independent of the percent ctDNA or allele fraction.
**Relevance**
The total number of alterations found on a liquid biopsy may be a marker of more aggressive tumor biology and has the potential to become a clinically meaningful, tissue-agnostic biomarker for use in advanced cancers and warrants additional testing in a prospective manner.

## MATERIALS AND METHODS

### Patient Data

Overall, 418 consecutive eligible patients at the University of California San Diego who had NGS (54 to 73 genes; Guardant Health, Redwood City, CA) performed on ctDNA derived from liquid (blood) biopsies were analyzed. Eligible patients included those who had solid tumor malignancies, never received immunotherapy treatment, and were evaluable for clinical correlations, including overall survival (OS) from ctDNA collection date. Patients had advanced/metastatic (stage IV) disease (except for patients with CNS tumors) at the time of ctDNA analysis. Immunotherapy-treated patients were omitted because a correlation with blood or tissue tumor mutational burden has been associated with better immunotherapy response and might therefore alter the survival curve.^19,20^ Patients with amplifications only in ctDNA were omitted because the %ctDNA for amplifications could not be determined. In addition to OS evaluation, patients’ data were also collected and analyzed for %ctDNA; the alteration with the highest allele fraction was calculated from all alterations, including variants of unknown significance (VUSs), total number of VUSs, and total number of alterations (which included VUSs). Percent ctDNA was evaluated as a continuous variable as well as using a cut-point of ≥ 5%, because this threshold had been found to be significant in prior studies.^10^ All studies and analyses were performed in accordance with the ethical guidelines of the Declaration of Helsinki and the Belmont Report per a University of California San Diego, Institutional Review Board-approved protocol (ClinicalTrials.gov identifier: NCT02478931) and the investigational treatment protocols for which the patients gave written consent.

### ctDNA Sequencing

Sequencing was performed by a Clinical Laboratory Improvement Amendments–certified and College of American Pathologists–accredited clinical laboratory, Guardant Health (http://www.guardanthealth.com). The Guardant360 (54-to-73 gene) panel identifies characterized and VUS tumor-related genomic alterations within cancer-related genes. All values for the total number of ctDNA alterations and the number of VUSs were corrected for the length (kilobase pairs [kbp]) of DNA sequenced based on the date sequencing was performed and multiplied by 100 (Appendix Table A1). All data were analyzed from the time of ctDNA collection from plasma (two 10-mL blood tubes). This ctDNA assay has a sensitivity and specificity of > 85% and > 99.9999%, respectively, for detection of single-nucleotide variants in tumor tissue of patients with advanced cancer.^21^

### Statistical Analysis

Statistical analysis was performed by P.R. Hazard ratios (HRs) for survival were calculated by comparing OS above and below cutoffs and performed from the time of ctDNA collection; dichotomization for each variable (ie, total number of alterations, total number of VUSs, %ctDNA) was performed at the median. Survival analyses were calculated by Kaplan-Meier analysis using log-rank (Mantel-Cox) test to generate *P* values, HRs, and CIs. Linear regressions were performed using the least squares method. Multivariate analyses were conducted using the Wald χ^2^ test from a Cox proportional hazards model that included all variables with *P* ≤ .05 in univariate analyses (ie, sex, age, total number of alterations, %ctDNA), with the exception of VUSs because these alterations are already encompassed within the total number of alterations variable. Patients alive at the time of last follow-up were censored at that date. Associations between %ctDNA and total number of alterations were determined using Spearman’s rank-order correlation. Bootstrapping using random sampling with replacement (N = 1,000 bootstrap samples) and multiple logistic regression analysis were performed, permitting the data of the sample study to be used as a surrogate for a larger population to validate the model. This method can be used when the sample size is too small to be split into training and validation sets and there is no independent cross-validation cohort, as was the case in our study.^22^ Statistical analyses were carried out using Prism version 7.0 (GraphPad, San Diego, CA) and R version 3.5 (R Foundation for Statistical Computing, Vienna, Austria).

## RESULTS

### Patient Characteristics

This study included 418 patients who had NGS performed on plasma-derived ctDNA and did not receive immunotherapy treatment. The median age at diagnosis was 60 years (range, 14-92 years) and the number of men (n = 191/418; 46%) and women (n = 227/418; 54%) were balanced. The most common tumor types included GI (n = 173/418; 41.4%), thoracic (n = 94/418; 22.5%), CNS (n = 51/418; 12.2%), and others (n = 100/418; 23.9%; Table 1). After correcting for the kbp length of DNA sequenced for each sample, the median total number of ctDNA alterations (including VUSs) per patient was 1.46 (range, 0-78.8); the median total number of VUS alterations per patient was 0.66 (range, 0-64.2); and the median %ctDNA was 0.4% (range, 0%-80.3%; Table 1). Among patients with GI tumors, the median total number of ctDNA alterations was 1.46 (range, 0-78.8), and the median %ctDNA was 0.5% (range, 0%-75%; Table 2).

**TABLE 1.**
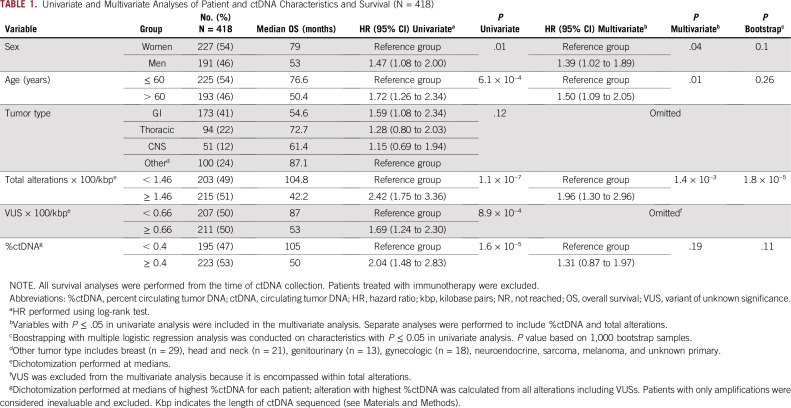
Univariate and Multivariate Analyses of Patient and ctDNA Characteristics and Survival (N = 418)

**TABLE 2.**
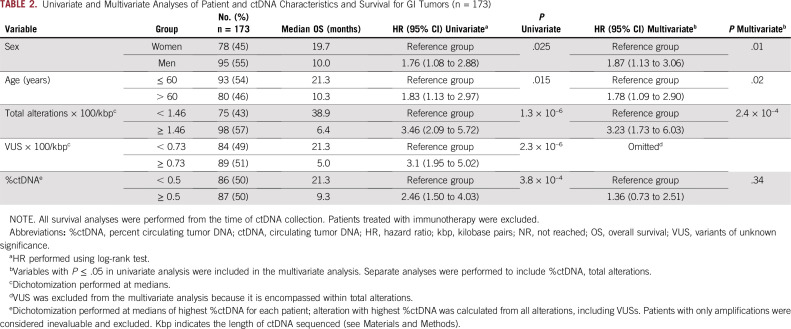
Univariate and Multivariate Analyses of Patient and ctDNA Characteristics and Survival for GI Tumors (n = 173)

### Factors Correlating With Survival in Univariate Analysis

The following factors showed significant correlations with poorer survival in univariate analysis: sex, older age (dichotomized at the median of 60 years), higher total number of alterations/kbp DNA (dichotomized at the median of 1.46), greater number of VUS alterations/kbp DNA (dichotomized at the median of 0.66), and higher %ctDNA (dichotomized at the median of 0.4%; Table 1). Tumor organ of origin was not found to be significantly correlated with differences in survival.

**FIG 1.**
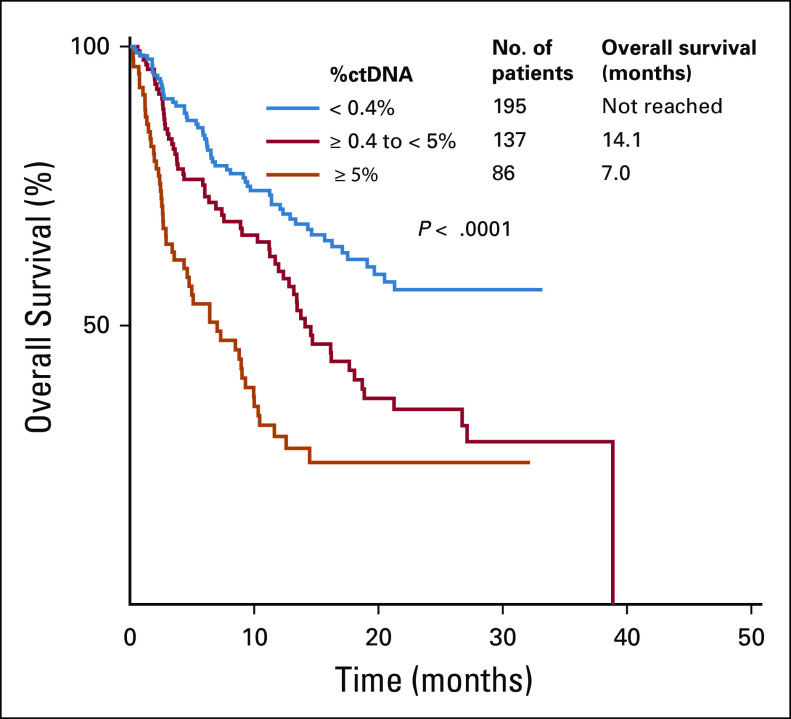
Overall survival from circulating tumor DNA (ctDNA) collection according to percent ctDNA (%ctDNA; N = 418). Low to intermediate %ctDNA was dichotomized at the median of 0.4%. Intermediate to high %ctDNA was dichotomized at 5% because it had been found to be significant in prior studies.^10^ The %ctDNA for each patient was calculated using the alteration with the highest allele fraction, including variants of unknown significance

**FIG A1.**
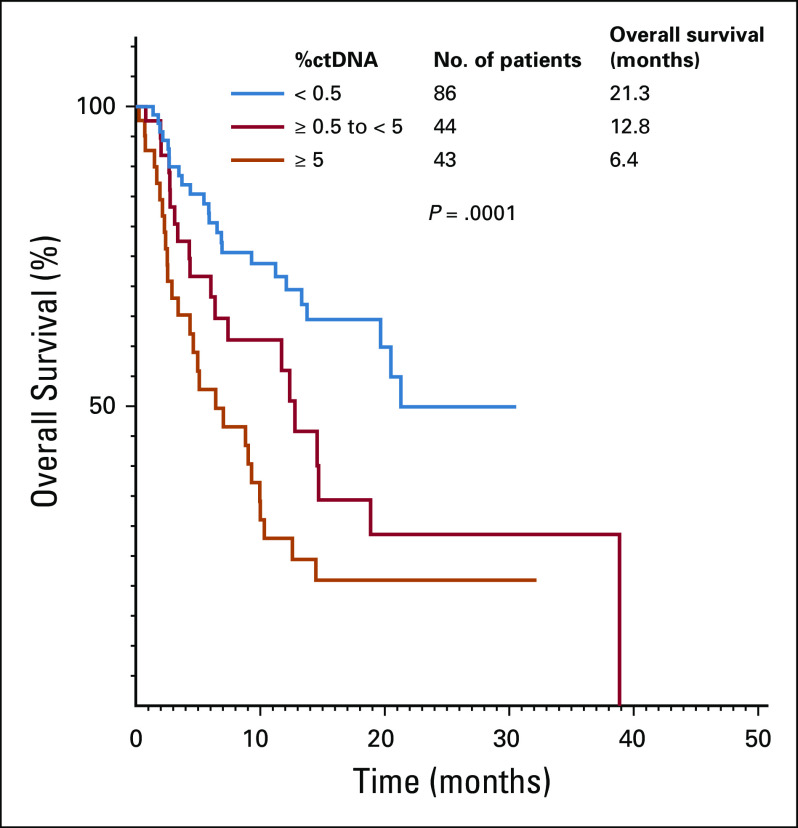
Overall survival from circulating tumor DNA (ctDNA) collection according to percent ctDNA (%ctDNA), for the GI patients only (n = 173). Low to intermediate %ctDNA was dichotomized at the median of 0.5%. Intermediate to high %ctDNA was dichotomized at 5% because it had been found to be significant in prior studies.^10^ The %ctDNA for each patient was calculated using the alteration with the highest allele fraction, including variants of unknown significance.

Patients with %ctDNA greater than or equal to the median of 0.4% had inferior OS compared with those with less than 0.4% (HR, 2.04; 95% CI, 1.48 to 2.83; *P* < .0001; Table 1). Furthermore, patients with a high %ctDNA (≥ 5%) had a significantly shorter OS compared with those who had an intermediate (≥ 0.4% to < 5%) or low (< 0.4%) value (median, OS 7.0 *v* 14.1 *v* not reached months, respectively; *P* < .0001; Fig 1). Among patients with GI tumors, those with %ctDNA greater than or equal to the GI median of 0.5% had worse survival outcomes (HR, 2.46; 95% CI, 1.50 to 4.03; *P* < .0001; Table 2; Appendix Fig A1).

**FIG 2.**
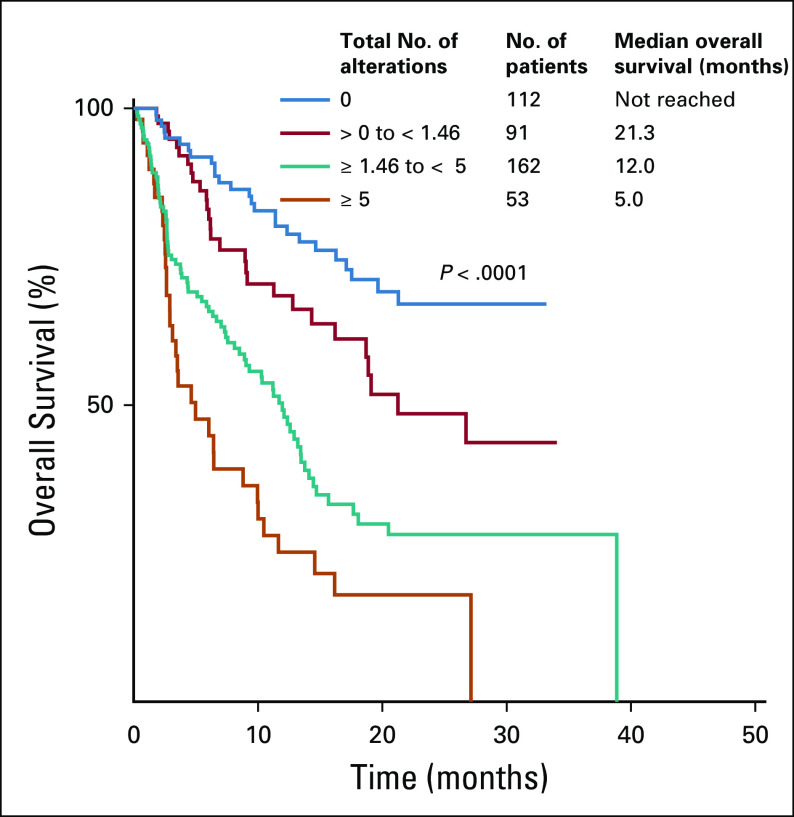
Overall survival from the date of circulating tumor DNA collection according to total alterations, including variants of unknown significance (N = 418). Low to intermediate number of alterations was dichotomized at the median of 1.46 alterations.

**FIG A2.**
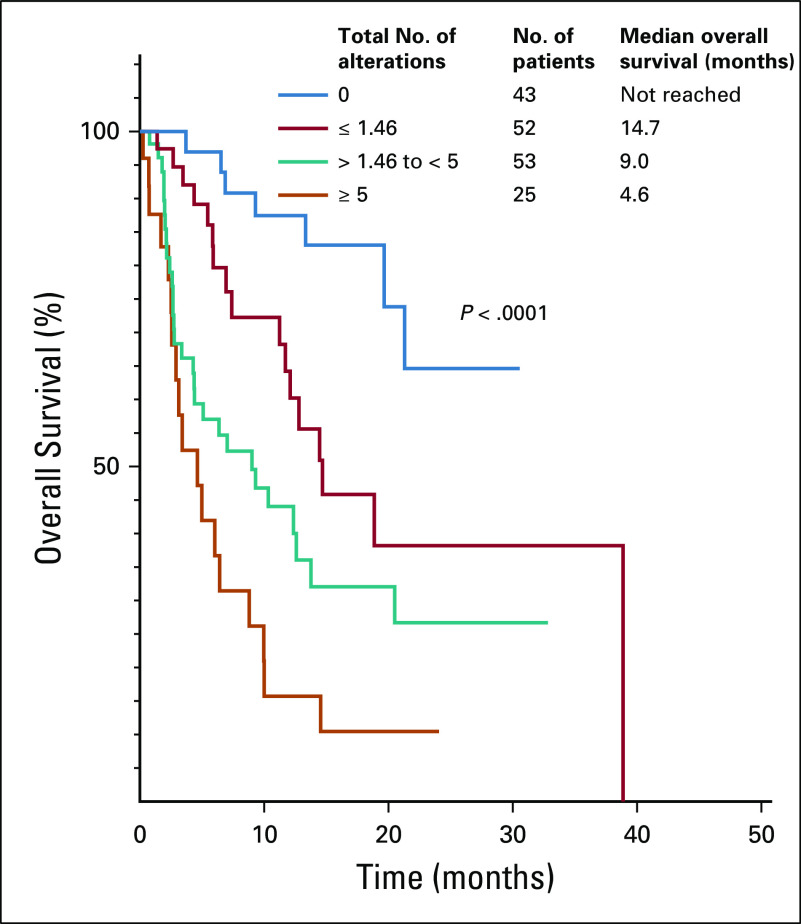
Overall survival from circulating tumor DNA collection according to total alterations, including variant of unknown significance for the GI patients only (n = 173). Low to intermediate number of alterations was dichotomized at the median of 1.46.

**FIG A3.**
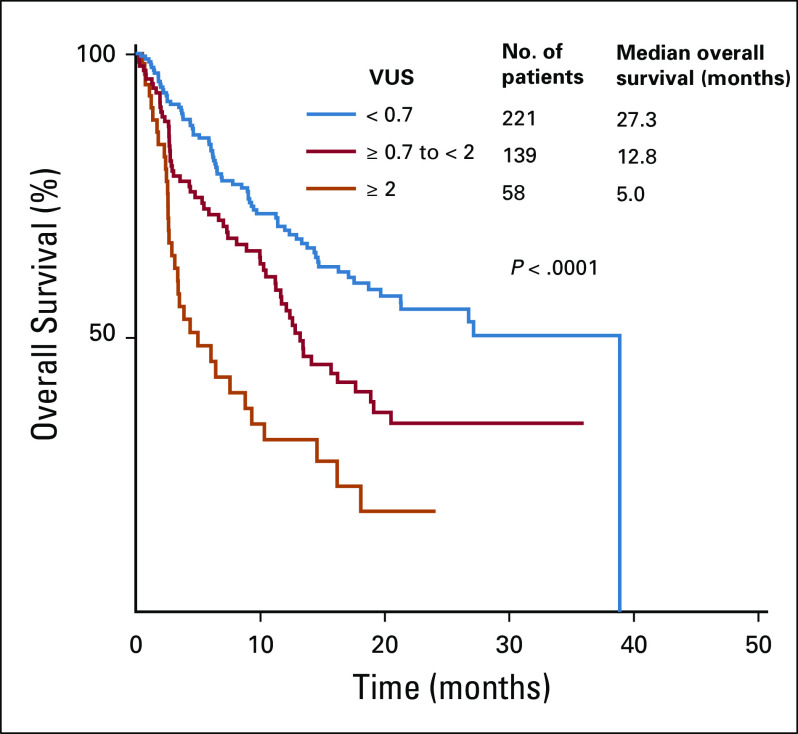
Overall survival from the date of circulating tumor DNA collection according to variant of unknown significance (VUS) (N = 418). Dichotomization for low to intermediate number of alterations was performed at the median of 0.7.

Likewise, patients with ≥ 1.46 total alterations/kbp DNA had statistically inferior OS compared with those who had less than the median of 1.46 total alterations/kbp DNA (HR, 2.42; 95% CI, 1.75 to 3.36; *P* < .0001; Table 1). Patients with a high (≥ 5) total number of alterations/kbp DNA had significantly shorter OS compared with those who had intermediate (≥ 1.46 to < 5), low (< 1.46), or no alterations (median OS, 4.6 *v* 11.7 *v* 21.3 *v* not reached months, respectively; *P* < .0001; Fig 2). In the subset of patients with GI tumors, patients with greater than or equal to the median of 1.46 total alterations/kbp had worse survival outcomes (HR, 3.46; 95% CI, 2.09 to 5.72; *P* < .0001; Table 2; Appendix Fig A2). Also, a higher number of VUS alterations/kbp DNA (≥ 0.66) was associated with worse OS compared with those with a lower number (< 0.66) of VUS alterations/kbp (HR, 1.69; 95% CI, 1.24 to 2.30; Table 1; Appendix Fig A3).

### Correlation Between %ctDNA and Total Number of Alterations

**FIG 3.**
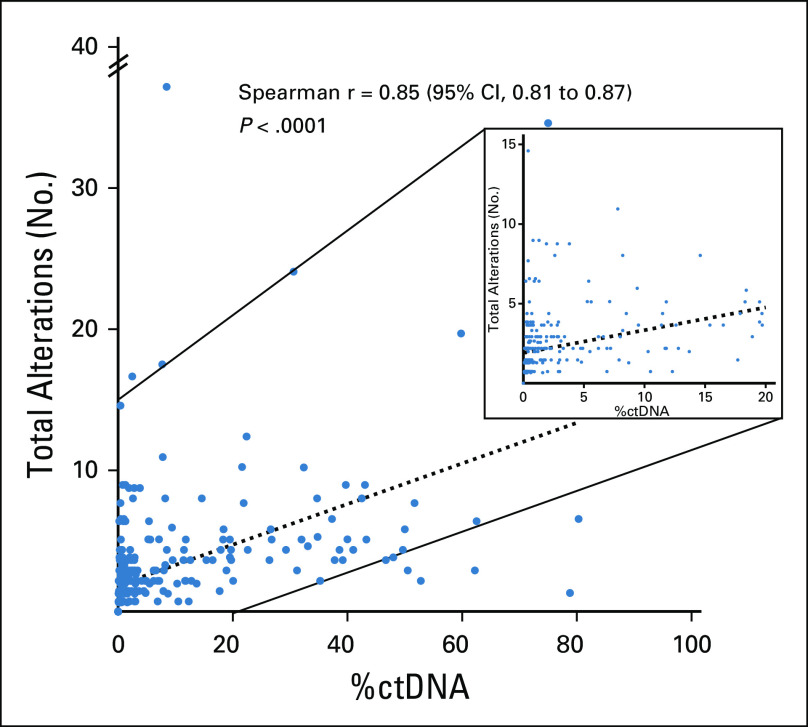
Spearman correlation between percent circulating tumor DNA (%ctDNA) and total number of alterations (N = 418).

**FIG A4.**
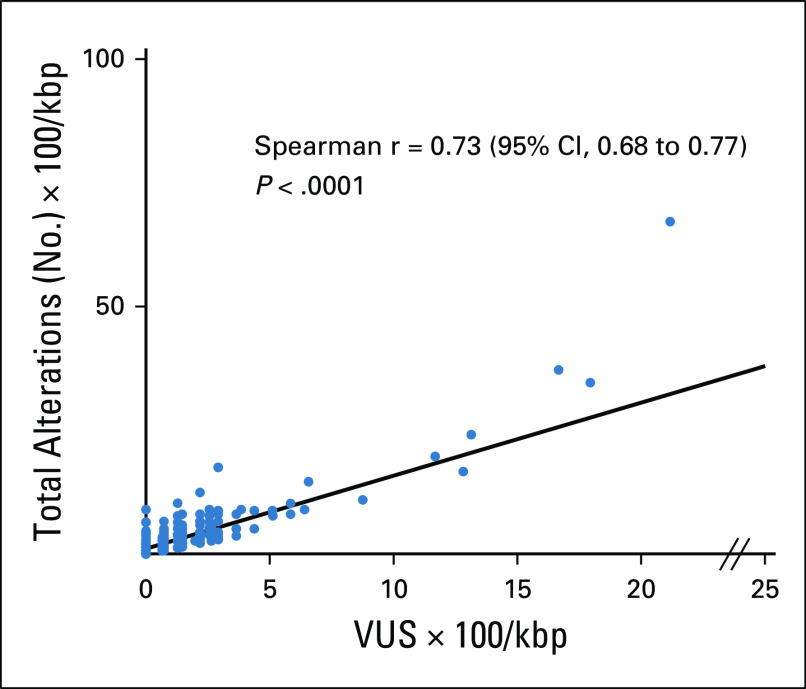
Spearman correlation between variant of unknown significance (VUS) and total alterations (N = 418).

The following ctDNA variables showed significant correlations with one another: the %ctDNA and the total number of alterations tended to increase together (r = 0.85; 95% CI, 0.81 to 0.87; *P* < .0001; Fig 3), and the number of VUS alterations and total number of alterations tended to increase together (r = 0.73; 95% CI, 0.68 to 0.77; *P* < .0001; Appendix Fig A4). To evaluate the influence of patients who had no detectable alterations (n = 112), we performed a sensitivity analysis removing these patients from the correlation calculations and found that there was still a significant (albeit attenuated) correlation between %ctDNA and total number of alterations (r = 0.61; *P* < .0001), as well as VUS and total number of alterations (r = 0.60; *P* < .0001).

### Factors Correlating With Survival in Multivariate Analysis

After accounting for sex, age, total number of alterations, and %ctDNA, a multivariable Cox proportional hazard regression model showed that age, sex, and the total number of alterations were independently prognostic for survival (Table 1). Specifically, patients with a high number of alterations (≥ 1.46) compared with those with fewer alterations (< 1.46) had worse OS (HR, 1.96; 95% CI, 1.30 to 2.96; *P* = .0014). Although statistically significant in univariate analysis, higher %ctDNA (≥ 0.4%) was not predictive of poorer survival compared to those with lower %ctDNA (< 0.4%; HR, 1.31; 95% CI, 0.87 to 1.97; *P* = .19) in multivariate analysis (Table 1). We also analyzed the subset of patients with GI tumors and found similar results (Table 2). Although univariate analyses of the GI subset of patients showed that both a high number of alterations (≥ 1.46) and higher %ctDNA (≥ 0.5%) had prognostic value, only the total number of alterations (HR, 3.23; 95% CI, 1.73 to 6.03; *P* < .0001), not the %ctDNA (HR, 1.36; 95% CI, 0.73 to 2.51; *P* = .34), was associated with worse outcomes in the multivariate model (Table 2).

### Analysis With Bootstrapping Method

Bootstrapping with multiple logistic regression was performed on all variables with *P* ≤ .05 in univariate analysis, which included sex, age, total number of alterations, and %ctDNA. Among these characteristics, only total number of alterations was significantly associated with survival (*P* < .0001; Table 1).

## DISCUSSION

Liquid biopsies have been incorporated into clinical practice as a means to obtain noninvasive molecular profiling to identify specific oncogenic driver mutations or other alterations that can guide treatment selection. In this study, we evaluated the relationship between the total number of alterations and the %ctDNA detected by liquid biopsy and survival outcomes in 418 patients with advanced cancers. The objective was to explore the potential prognostic value of blood-based NGS. It should be noted that we adjusted for changes in sequencing length by correcting the total number of alterations and VUSs for the amount of DNA sequenced. In addition, we intentionally excluded patients who subsequently received immunotherapy treatment, because several studies have suggested that the use of immune checkpoint inhibitors may alter the survival curve in patients with increased tumor mutational burden.^19,20,23^

We demonstrate that both the total number of alterations and the %ctDNA have prognostic value and correlate with one another, but only an intermediate to high (≥ 1.46) total number of alterations/kbp (and not high %ctDNA) was independently associated with worse survival outcomes in multivariate analysis in patients with GI tumors (Table 2), as well as in patients with a diverse group of advanced cancers (Table 1). These findings were then internally validated using bootstrap resampling. Our findings suggest that more alterations per kbp DNA detected in plasma may be a better indicator of more aggressive tumor biology and therefore poorer survival than %ctDNA. It is also plausible that the higher number of alterations and accompanying aggressive biology results in a higher tumor burden that yields a higher %ctDNA (rather than vice versa).

A strength of our study is that we used sequencing technology that allows for the detection of %ctDNA at a low level with high sensitivity and high specificity.^21,24^ In comparison, some prior studies have used low-depth sequencing, which is less capable of detecting ctDNA. As a result, these studies were only able to conclude that the presence of ctDNA was associated with worse outcomes compared with the absence of detectable ctDNA.^14,18,25,26^ Indeed, Yang et al^27^ proposed that the presence or absence of ctDNA should be added to the TNM staging classification of tumors because it has diagnostic, therapeutic, and prognostic value. When greater depth of ctDNA sequencing was used, prior studies have reported that %ctDNA is correlated with worse survival and also with increased tumor volume.^10,11,14^ We also demonstrated that %ctDNA correlates with survival measured from the time of blood draw (Fig 1), which suggests that the association between %ctDNA and outcomes may be more reflective of tumor burden.

There are several limitations to our findings, given the retrospective nature of the analysis. Although our study used a relatively large sample of 418 patients, we included a diverse group of advanced cancers and, therefore, our findings may not be applicable to certain tumor types. Despite this, the variety of tumor types in our study may make our findings more generalizable across advanced cancers. However, we also performed the analyses on a cohort of 173 patients with GI tumors and found similar results (Table 2). In addition, 112 patients in this study had no detectable %ctDNA, which may be due to low disease burden or due to limitations of the ctDNA sequencing technique. It should also be noted that there was a different number of subgroups in the analysis of %ctDNA and number of ctDNA alterations; hence, the conclusion that the total number of alterations and %ctDNA have prognostic value and correlate with one another but that only the total number of alterations was independently associated with survival outcomes will need to be further examined and validated. Also, we do not know whether this patient population is comparable with those who were not analyzed for ctDNA because physicians chose not to perform the analysis or with patients who were lost to follow-up early and hence were inevaluable. Finally, patients had a diverse array of prior treatments, some of which could have confounded the results; patients treated with immunotherapy were excluded because cancers with higher mutational burden/number appear to do better on this modality.

In conclusion, to our knowledge, this is the first demonstration that the total number of alterations and %ctDNA are highly correlated and have prognostic value. Nevertheless, in multivariate analysis, only the total number of alterations was independently predictive of OS. Understanding the prognostic value of ctDNA is important in and of itself, but also has implications as a confounder, because ctDNA is being used as a predictive marker for the efficacy of drugs such as immunotherapy.^20^ To summarize, the total number of alterations has the potential to become a clinically meaningful, tissue-agnostic biomarker for use in advanced cancers and warrants additional testing in a prospective manner.

## Figures and Tables

**TABLE A1.**
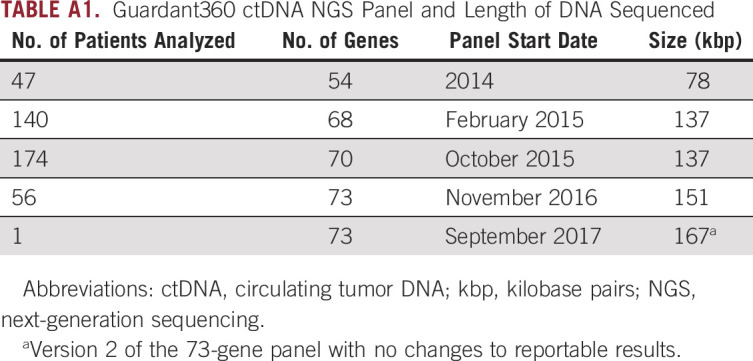
Guardant360 ctDNA NGS Panel and Length of DNA Sequenced
